# Assessment of Cidofovir for Treatment of Ocular Bovine Herpesvirus-1 Infection in Cattle Using an Ex-Vivo Model

**DOI:** 10.3390/v13102102

**Published:** 2021-10-18

**Authors:** Christopher R. Alling, Chin-Chi Liu, Ingeborg M. Langohr, Muzammel Haque, Renee T. Carter, Rose E. Baker, Andrew C. Lewin

**Affiliations:** 1 Department of Veterinary Clinical Sciences, School of Veterinary Medicine, Louisiana State University, Baton Rouge, LA 70803, USA; alling1@lsu.edu (C.R.A.); cliu@lsu.edu (C.-C.L.); reneecarter@lsu.edu (R.T.C.); baker3@lsu.edu (R.E.B.); 2 Department of Pathobiological Sciences, School of Veterinary Medicine, Louisiana State University, Baton Rouge, LA 70803, USA; ilangohr@lsu.edu (I.M.L.); mhaque2@lsu.edu (M.H.)

**Keywords:** Bovine herpesvirus-1, keratoconjunctivitis, ex-vivo corneal culture, cidofovir, nucleotide analogue antiviral agent

## Abstract

Bovine herpesvirus-1 (BoHV-1) infection contributes to keratoconjunctivitis, respiratory disease, and reproductive losses in cattle. The objective of this study was to determine the most appropriate ophthalmic antiviral agent for BoHV-1 inhibition using in-vitro culture and novel ex-vivo bovine corneal modeling. Half-maximal inhibitory concentrations of BoHV-1 were determined for cidofovir, ganciclovir, idoxuridine, and trifluridine via in-vitro plaque reduction assays. In-vitro cytotoxicity was compared amongst these compounds via luciferase assays. Trifluridine and cidofovir were the most potent BoHV-1 inhibitors in vitro, while trifluridine and idoxuridine were the most cytotoxic agents. Therefore, cidofovir was the most potent non-cytotoxic agent and was employed in the ex-vivo corneal assay. Corneoscleral rings (*n* = 36) from fresh cadaver bovine globes were harvested and equally divided into an uninfected, untreated control group; a BoHV-1-infected, untreated group; and a BoHV-1-infected, cidofovir-treated group. Virus isolation for BoHV-1 titers was performed from corneal tissue and liquid media. Histologic measurements of corneal thickness, epithelial cell density, and tissue organization were compared between groups. Substantial BoHV-1 replication was observed in infected, untreated corneas, but BoHV-1 titer was significantly reduced in cidofovir-treated (1.69 ± 0.08 × 10^3^ PFU/mL) versus untreated (8.25 ± 0.25 × 10^5^ PFU/mL, *p* < 0.0001) tissues by day 2 of culture. No significant differences in histologic criteria were observed between groups. In conclusion, cidofovir warrants further investigation as treatment for BoHV-1 keratoconjunctivitis, with future studies needed to assess in-vivo tolerability and efficacy.

## 1. Introduction

Bovine herpesvirus type 1 (BoHV-1) is a global pathogen of the *Varicellovirus* genus of the *Alphaherpesvirinae* subfamily that presents a significant economic and welfare burden to domestic ruminants and livestock producers. Known to be the causative agent of infectious bovine rhinotracheitis (IBR), it contributes to the Bovine Respiratory Disease Complex (BRDC, or “shipping fever”), which causes significant economic losses in beef production from reduced weight gain, mortality, and treatment expenditures [1,2] as well as reduced milk production in dairy cattle [3]. Additional manifestations of clinical disease include urogenital infection with abortion in adult cattle, thereby resulting in further economic losses from prolonged calving interval as well as encephalitis and enteritis in calves [1]. The virus is also thought to be involved in the development of infectious bovine keratoconjunctivitis (IBK, or “pinkeye”), a disease with significant associated costs from treatment expenditures and reduced production indices, such as average daily weight gain and weaning weight in beef production [4,5] as well as severe welfare consequences due to its painful and potentially blinding effects [6].

While *Moraxella bovis* has been established as the primary etiologic agent of IBK, BoHV-1 infection is thought to contribute more significantly to clinical signs of conjunctivitis than *M. bovis* [7,8]. Additionally, BoHV-1 may predispose individuals to *M. bovis* infection [8,9]. Specific clinical signs historically attributed to BoHV-1 infection in cattle include non-ulcerative keratoconjunctivitis with raised, white plaques representing lymphoid follicles of the bulbar and palpebral conjunctivae; chemosis (conjunctival swelling); corneal edema; and corneal neovascularization [6].

Significant efforts have been taken to investigate treatment of herpesvirus-associated keratoconjunctivitis with topical antiviral nucleoside analogue compounds in multiple domestic animal species [10,11,12,13,14,15,16,17,18,19]. Because herpesviruses establish latency within various tissues of host species, these treatments are generally considered virostatic and are intended to mitigate the severity and duration of clinical signs while preventing debilitating and potentially blinding sequelae, such as secondary septic keratitis, corneal sequestrum, eosinophilic keratitis, corneal fibrosis, or corneal perforation [20]. In-vitro antiviral assays against BoHV-1 have also been reported for selected nucleoside analogue agents [21,22,23,24,25,26], but a direct comparison of the most accessible and cost-effective antiviral agents is currently lacking for ruminant species.

In-vitro assays are proven methods for investigating novel applications of antiviral compounds for herpesvirus-associated ocular disease in several domestic animal species [10,11,12,13,16,17,18,19]. However, of increasing importance are ex-vivo culture systems utilizing biological specimens that retain normal tissue architecture to better approximate conditions encountered by infectious agents in vivo [27,28,29,30,31,32,33,34,35]. To the authors’ knowledge, an ex-vivo bovine corneal culture model for BoHV-1 assessment has not been previously described.

The objectives of this study were to compare the in-vitro efficacy of commonly available topical antiviral compounds (cidofovir, ganciclovir, idoxuridine, and trifluridine) against BoHV-1 to assess the relative cytotoxicity of these compounds to bovine tissues and to demonstrate the feasibility of a novel ex-vivo bovine cornea model for assessment of antiviral efficacy of BoHV-1-related keratoconjunctivitis. Based on previous evidence [21,23,24], we hypothesized that trifluridine would exhibit superior antiviral efficacy against BoHV-1 but would also exhibit increased cytotoxicity and that all other compounds would be well tolerated by bovine tissues.

## 2. Materials and Methods

### 2.1. In-Vitro Plaque Reduction Assay

Madin–Darby Bovine Kidney cells (MDBK (NBL-1), ATCC^®^ CCL-22TM) were cultured at 37 °C/5% CO_2_ to create confluent monolayers on 24-well, treated tissue culture plates (Fisherbrand, Waltham, MA, USA) in Dulbecco’s Modified Eagle Media (DMEM, Gibco, Waltham, MA, USA) containing 10% fetal bovine serum (FBS, Gibco) and 1% Penicillin-Streptomycin (PS, Gibco). MDBK cells were chosen because they are a readily available, reliable, and well-characterized cell line for propagation of BoHV-1 utilized in previous studies of antiviral efficacy [23,24]. Fifty plaque forming units (PFU) of BoHV-1 (Cooper Strain VR864TM, ATCC) in DMEM + 2% FBS + 1% PS were added to each well to infect the cells over 1 h. The supernatant from each well was then replaced with DMEM + 2% FBS + 1% PS + 1% methylcellulose (MC) containing either no drug (negative control) or an added dilution of the following antiviral agents: cidofovir (1-[[30]-3-hydroxy-2(phosphonomethoxy)propyl] cytosine, MedChemExpress, Monmouth Junction, NJ, USA), ganciclovir (9-[1,3-dihydroxy-2-propoxy-methyl]guanine, Acros Organics, Fair Lawn, NJ, USA), idoxuridine (1-[2-deoxy-β-d-ribofuranosyl]-5-iodouracil, Cayman Chemical, Ann Arbor, MI, USA), and trifluridine (2′-deoxy-5-trifluoromethyluridine, Acros Organics). A preliminary range of concentrations of 1–100 μM near the presumptive half maximal inhibitory concentration (IC_50_) for plaque number and plaque area was tested for all agents. For clarity, IC_50Number_ is defined as the concentration of antiviral compound required to reduce the number of plaques per well by 50% relative to the untreated control, whereas IC_50Area_ is defined as the concentration of antiviral compound required to reduce the average two-dimensional surface area of viral plaques in each well by 50% relative to the untreated control [19]. Additional tested drug concentrations were chosen based upon calculated inhibition from this initial range. All wells (negative controls and experimental drug concentrations) were performed with 4 replicates each. Upon addition of the drug-containing methylcellulose media, plates were incubated at 37 °C/5% CO_2_ for 48 h. The methylcellulose-containing media was removed, and the cells were fixed with methanol. After at least 30 min of incubation at −20 °C, methanol was decanted from the wells and replaced with crystal violet solution (0.5%, Fisher Scientific, Waltham, MA, USA) for 5 min at room temperature to stain the cells. Plaque number and area were measured using a light microscope (Olympus MVX10 Research Macro Zoom with Olympus DP72 digital camera system, Tokyo, Japan) and measurement software (Olympus CellSens). For plaque area measurement, 10 plaques per well were randomly chosen, and at least 8 vertices around the perimeter of each plaque were delineated using the CellSens “polygon” measurement tool. Data points for plaque number and area were plotted separately using a commercially available software (Microsoft Excel, Redmond, WA, USA) with regression lines of best fit used to calculate the half maximal inhibitory concentration (IC_50_) for plaque number and area of each drug via a curve fit method.

### 2.2. In-Vitro Cytotoxicity Assay

To compare the antiviral compounds employed in the in-vitro assays for cytotoxic effects in MDBK cells, a CellTiter-Glo^®^ kit (Promega, Madison, WI, USA) was used according to manufacturer recommendations. Briefly, trypsinized MDBK cells (grown at 37 °C/5% CO_2_) in DMEM + 10% FBS/1% PS were transferred to 96-well tissue culture plates (Corning Costar, Cambridge, MA, USA). After incubation at room temperature for 1 h, growth medium was decanted and replaced with DMEM + 2% FBS/1% PS containing 1X, 2X, 3X, 5X, or 10X the IC_50Area_ values established for each antiviral compound (cidofovir, ganciclovir, idoxuridine, and trifluridine) in the plaque reduction assay. Negative control wells containing MDBK cells in media without added antiviral compounds and separate blank wells containing only liquid media without cells were included on each plate. Plates were incubated at 37 °C/5% CO_2_ for 48 h and were then transferred to room temperature for 30 min immediately prior to the assay. CellTiter-Glo^®^ reagent (containing a patented variant of luciferase enzyme) was added to each well of MDBK cells, and cell lysis was induced using an orbital shaker at room temperature for 2 min. Following lysis, plates were incubated at room temperature for 10 min before luminescence was measured with a microplate reader (Synergy HTX, BioTek, Winooski, VT, USA). Eight replicates were assessed for all combinations of antiviral compounds and concentrations. Cytotoxicity was expressed as relative luminescence (RL), which is calculated as the mean luminescence of a given antiviral compound/concentration divided by the mean luminescence of the plate negative control.

### 2.3. Ex-Vivo Corneal Assay

#### 2.3.1. Corneal Tissue Culture

**Figure 1:**
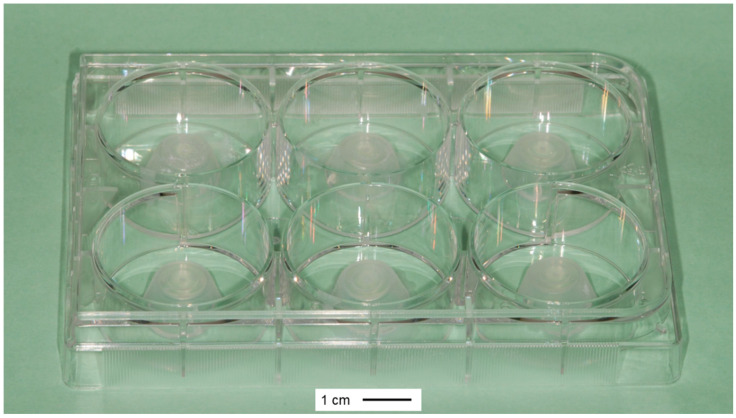
Culture plate for ex-vivo corneal assay featuring plastic pedestal corneal conformers.

A novel ex-vivo bovine cornea model was developed and utilized with modifications of previously described protocols for equine, feline, and canine corneal tissues [29,30,31]. Prior to the first day of the experiment, plastic pedestal conformers for corneal support during tissue culture were created by removing the rounded ends of 6 mL plastic Luer lock syringe casings (Covidien, Dublin, Ireland) and cutting them to ½ inch (1.27 cm) height. Each resulting pedestal was secured centrally (concave-side down) within the wells of 6-well tissue culture plates (Corning Costar) using cyanoacrylate adhesive (Krazy Glue^®^, Elmer’s Products, Westerville, OH, USA). This design was intended to preserve the normal bovine corneal curvature radius and maintain three-dimensional corneal shape (Figure 1). The plates were gas-sterilized with ethylene oxide prior to use.

Cadaver bovine globe specimens were obtained from a commercial vendor (Animal Technologies, Tyler, TX, USA) via refrigerated, overnight shipping and were processed immediately upon receipt. The globes were inspected grossly prior to processing to ensure no corneal epithelial or stromal lesions were present. Each globe was transferred to a sterile petri dish (Corning Falcon) and was rinsed with three cycles of 2% povidone iodine solution and sterile phosphate buffer saline (PBS, Gibco). A #11 Bard Parker^®^ blade (Aspen Surgical, Caledonia, MI, USA) was used to create a penetrating stab incision through the sclera 2–3 mm posterior to the limbus; the incision was extended circumferentially at a consistent distance from the limbus using Mayo scissors to create a corneoscleral ring (CSR). Each CSR was rinsed again with three cycles of povidone iodine solution and PBS before further processing in one of three experimental groups (*n* = 12 CSRs per group): an uninfected, untreated negative control group; a BoHV-1-infected, untreated group (I); and a BoHV-1-infected, cidofovir-treated group (I+T). CSRs of the control group were immediately placed epithelium side up in individual wells of the previously described gas-sterilized 6-well conformer plates containing 5 mL of EpiLife^®^ medium (Gibco) + 10% FBS + 1% PS per well; plates were then secured to an orbital rocker with 2-dimensional axial motion at 7 revolutions per minute based upon the reported range of interblink intervals for dairy cattle [36] and were incubated at 37 °C/5% CO_2_ until further processing. CSRs of the I group were infected by placing them epithelium side down in unmodified 6-well plates without conformers, each well containing 5 mL EpiLife^®^ medium + 2% FBS + 1% PS + 3 × 10^5^ PFU BoHV-1 (Cooper Strain VR864TM, ATCC). These plates were incubated for 1 h at 37 °C/5% CO_2_ on an orbital rocker before rinsing each CSR with 3 cycles of PBS and transferring them to 6-well conformer plates with 5 mL fresh supplemented EpiLife^®^ medium per well and incubating them as per the control group above. CSRs of the I+T group were infected identically to the I group, but after PBS rinsing, they were placed into 6-well conformer plates containing 5 mL EpiLife^®^ medium + 10% FBS + 1% PS + 18 μM (10X IC_50Area_) cidofovir per well and incubated as per the control group above.

CSRs were further processed at the following timepoints post-receipt: 2 h (day 0), 26 h (day 1), 50 h (day 2), and 74 h (day 3). At each interval, three CSRs from each group were removed from culture and transferred to sterile petri dishes. EpiLife^®^ medium was collected from the corresponding wells and pooled per group/timepoint into 50-mL conical tubes, which were stored at −80 °C for subsequent BoHV-1 titer measurement. A 4-mm Baker punch biopsy (Integra Miltex, Princeton, NJ, USA) was used to harvest a corneal button from the perilimbal cornea of each CSR using a unidirectional clockwise cutting motion; the buttons were pooled per group/timepoint into a single RINO^®^ tube (Next Advance, Troy, NY, USA) containing 1 mL DMEM, 3 stainless steel spherical beads (3.2 mm, Next Advance), and 3 stainless steel UFO beads (3.5 mm diagonal, Next Advance). The buttons were then homogenized using a Bullet Blender 24 Gold (Next Advance) according to manufacturer instructions (4 °C, speed “8”, 15 min total) and were stored at −80 °C for subsequent BoHV-1 titer measurement. A histology razor blade was used to hemisection the corneas. The halves from which the corneal buttons were harvested were discarded. One transverse section extending from the perilimbal sclera to the axial cornea was collected from each of the remaining halves and placed into tissue cassettes. The samples were fixed in 10% neutral buffered formalin (StatLab, McKinney, TX, USA) for histopathological assessment.

#### 2.3.2. BoHV-1 Titer Measurement

MDBK cells were grown at 37 °C/5% CO_2_ to confluent monolayers on 12-well, treated tissue culture plates (Fisherbrand) in DMEM + 10% FBS + 1% PS for standard virus isolation titer measurement. Pooled liquid culture media and corneal button samples were subjected to 3 freeze-thaw cycles and centrifuged at 1600 rpm × 4 min. The supernatant from each sample was collected, and serial 10-fold dilutions of 10^−1^–10^−6^ were prepared. Following decanting of DMEM from the plate wells, 400 μL of each dilution were added to separate wells (2 replicates of each dilution per plate). Cells were incubated with the dilutions at room temperature on an orbital rocker for 1 h before the supernatants were replaced with DMEM + 2% FBS + 1%PS + 1% MC. Plates were then incubated for 48 h at 37 °C/5% CO_2_ and stained with crystal violet as described above per the antiviral plaque reduction assay. Plaque number was determined at the lowest countable dilution factor and averaged between the 2 replicates per plate; BoHV-1 titers were then calculated and expressed as PFU/mL.

#### 2.3.3. Corneal Histopathology

**Figure 2:**
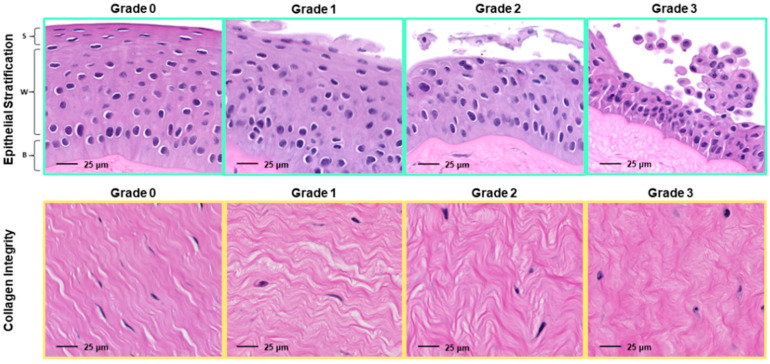
Semiquantitative histopathology grading scheme for bovine corneal sections. Epithelial stratification:0 = clear distinction of basal, wing, and squamous cell layers; 1 = weak disorganization with minor squamous separation; 2 = moderate disorganization with moderate squamous separation; 3 = marked disorganization with significant squamous separation and degenerate basal cell attachments. Basal (B), wing (W), and squamous (S) cell layers are indicated in the Grade 0 image. Stromal organization: 0 = no stromal disorganization; 1 = weak stromal disorganization; 2 = moderate stromal disorganization; 3 = marked stromal disorganization. All images are photomicrographs magnified to 400X.

Formalin-fixed corneal sections were routinely processed for histological evaluation with hematoxylin and eosin (H&E) staining. Corneal sections (*n* = 3 per group/timepoint) were evaluated at 5 locations (perilimbal, far-paraxial, mid-paraxial, near-paraxial, and axial) for objective (epithelial thickness, stromal thickness, epithelial cell density) and semiquantitative (epithelial stratification, stromal organization) measures of corneal health. Therefore, for each parameter, there were 15 measurements per group/timepoint, with 180 total tissue sites evaluated altogether. Objective parameters were measured using Aperio ImageScope (Leica Biosystems, Wetzlar, Germany) following slide digitization with a NanoZoomer slide scanner (Hamamatsu, Iwata, Japan). Semiquantitative parameters were graded by a board-certified veterinary pathologist (IML), a board-certified veterinary ophthalmologist (ACL), and a veterinary resident training in comparative ophthalmology (CRA) via light microscopy using a previously described scale employed in an equine ex-vivo corneal tissue model [31]—the reader is referred to Figure 2 for a complete description of the grading scheme. Observers were masked to group/timepoint prior to grading. Semiquantitative scores were averaged between observers prior to statistical analysis.

### 2.4. Statistical Analysis

Data analyses were performed using JMP Pro 15.0.0 (SAS Institute Inc., Cary, NC, USA). Experiment continuous values were assessed with an analysis of variance (ANOVA) model with treatment, day, and their interaction as the fixed effects. Titer values were log-transformed. Assumptions of these models (linearity, normality of residuals, and homoscedasticity of residuals) and influential data points were assessed by examining standardized residual and quantile plots. When a fixed effect was detected, Tukey post-hoc comparisons were performed with least square means for the effect. H&E grading was evaluated with a Friedman rank test within each day or each treatment against treatment or day. Significance was set at *p* < 0.05.

## 3. Results

### 3.1. In-Vitro Plaque Reduction Assay

The IC_50_ values for plaque number and area are listed in Table 1 along with the corresponding number of concentrations tested for each drug. Trifluridine was the most potent inhibitor of BoHV-1 for both categories, but marked visible cytotoxicity (MDBK cell rounding with poor adhesion and monolayer degradation) was observed at concentrations as low as 0.001 μM. For this reason, a relatively small number of concentrations of trifluridine (*n* = 6) were tested. Cidofovir was the second most potent inhibitor of BoHV-1 for both categories and did not exhibit visible cytotoxicity for any of the concentrations tested. There was a relatively large difference in antiviral potency between cidofovir and idoxuridine as well as between idoxuridine and ganciclovir.

### 3.2. In Vitro Cytotoxicity Assay

**Figure 3:**
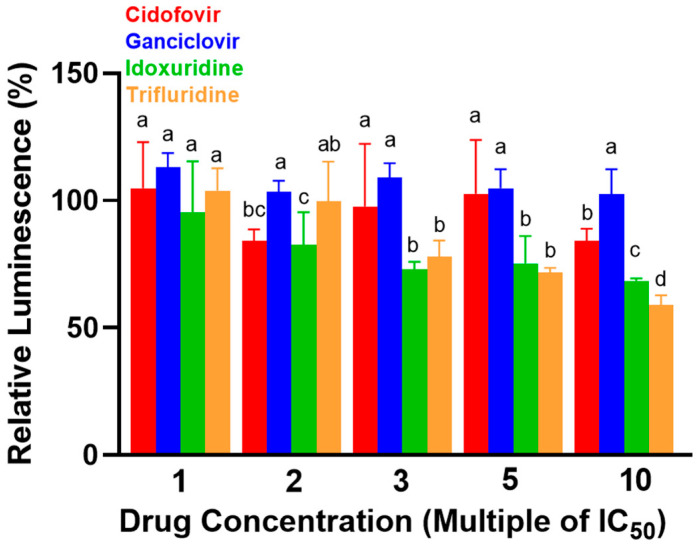
In-vitro comparison of nucleoside analogue antiviral agent cytotoxicity to Madin–Darby Bovine Kidney (MDBK) cells via CellTiter-Glo^®^ assay. Relative luminescence (RL) is correlated with cellular ATP concentration; lower relative luminescence therefore indicates greater cytotoxicity. Lowercase letter labels “a–d” refer to statistical comparisons of RL amongst antiviral compounds—columns connected by the same letter labels are not significantly different (*p* > 0.05).

The results of the CellTiter-Glo^®^ assay are depicted in Figure 3. No significant differences in mean relative luminescence (±standard error) were observed between the four antiviral compounds when tested at their 1X IC_50Area_ concentrations; at 2X IC_50Area_, cidofovir was significantly more cytotoxic than ganciclovir (RL 84.2 ± 4.5% vs. 103.5 ± 4.2%, respectively; *p* = 0.0202) but did not have significantly different cytotoxicity from either trifluridine or idoxuridine (RL 99.9 ± 3.9% (*p* = 0.0626) and 82.7 ± 3.9% (*p* = 0.9944), respectively). However, increasing the concentration of the compounds to higher multiples of the IC_50Area_ resulted in a somewhat variable but overall consistent separation between the two agents that were associated with less cytotoxicity (cidofovir and ganciclovir) and the two agents associated with greater cytotoxicity (idoxuridine and trifluridine). For example, at 3X IC_50Area_ cidofovir and ganciclovir did not have significantly different cytotoxicity (RF 97.6 ± 4.7% vs. 108.9 ± 4.7%, respectively (*p* = 0.3539)), but both were significantly less cytotoxic than either trifluridine or idoxuridine (RF 77.9 ± 4.7% and 73.1 ± 5.1%, respectively; *p*-values for individual comparisons not included). A similar pattern was observed at 5X IC_50Area_. At 10X IC_50Area_, cidofovir was again significantly more cytotoxic than ganciclovir (RF 84.1 ± 2.3% and 102.4 ± 2.2%, respectively (*p* < 0.0001)) but was significantly less cytotoxic than both trifluridine and idoxuridine (RF 58.9 ± 2.0% (*p* < 0.0001) and 68.4 ± 2.0% (*p* = 0.0002), respectively). Given that cidofovir was the most potent inhibitor of BoHV-1 amongst the less cytotoxic antiviral agents, this drug was selected as the sole antiviral agent to be tested in the ex-vivo branch of the study.

### 3.3. Ex-Vivo Corneal Assay

#### 3.3.1. BoHV-1 Titer Measurement

**Figure 4:**
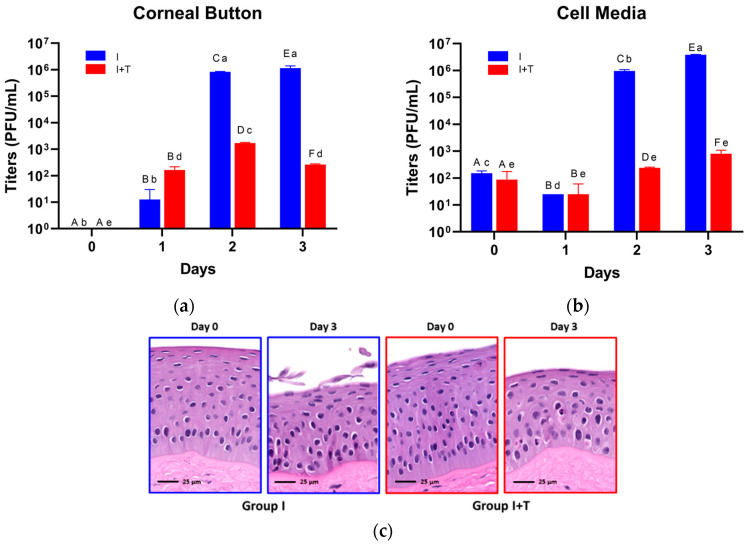
Homogenized corneal button (**a**) and liquid EpiLife^®^ cell media (**b**) BoHV-1 titers for ex vivo bovine corneal culture. I = BoHV-1-infected, untreated group; I+T = BoHV-1-infected, cidofovir-treated group. Columns connected by the same letter labels are not significantly different (*p* > 0.05). Uppercase letters correspond to within-day comparisons. Lowercase letters correspond to within-treatment comparisons. (**c**) Representative photomicrographs from histologic specimens of the I and I+T groups on days 0 and 3 depicting corneal epithelial changes over the duration of culture. In the I group, attenuation of the epithelium with cellular condensation, disorganization of epithelial stratification, heterogenous nuclear chromatin, and exfoliation of the squamous epithelium are seen on day 3. In the I+T group, attenuation of the epithelium, cellular condensation, and multiple eosinophilic cytoplasmic inclusions (suggesting cell death) are seen.

BoHV-1 titer values for both liquid corneal culture media and corneal button homogenates are depicted in Figure 4. No BoHV-1 was recovered from control corneal buttons at any time point. Similarly, no BoHV-1 was recovered from corneal buttons on day 0 (BoHV = 0 ± 0.0 PFU/mL, BoHV + Cid = 0.0 ± 0.0 PFU/mL, *p* = 1.0000—Figure 4A). By day 1, BoHV-1 had replicated in corneal tissues in both groups, but titers were not significantly different between groups (I = 12.5 ± 12.5 PFU/mL, I+T = 162.5 ± 37.5 PFU/mL, *p* = 0.1677). However, by day 2, titers had risen significantly in both groups relative to the previous day (I = 8.25 ± 0.25 × 10^5^ PFU/mL, *p* = 0.0016; I+T = 1.69 ± 0.08 × 10^3^ PFU/mL, *p* = 0.0006), with the I group exhibiting a significantly higher titer than that of the I+T group (*p* < 0.0001). By day 3, the I group titer remained significantly higher than that of the I+T group (1.14 ± 0.17 × 10^6^ PFU/mL vs. 262.5 ± 2.5 PFU/mL, respectively; *p* = 0.0004). Moreover, while the day 3 I group titer was not significantly different from the previous day (*p* = 0.9911), the day 3 I+T group titer fell significantly relative to the previous day (*p* = 0.0015). Liquid corneal media titers exhibited a similar pattern (Figure 4B), with no BoHV-1 recovered from control samples at any time point and insignificant differences between the I and I+T groups measured on days 0 (*p* = 0.4354) and 1 (*p* = 0.5847) that shifted to significantly higher titers in the I group relative to the I+T group on days 2 (9.63 ± 0.88 × 10^5^ PFU/mL vs. 237.5 ± 12.5 PFU/mL, respectively; *p* = 0.0002) and 3 (3.84 ± 0.11 × 10^6^ PFU/mL vs. 800 ± 200 PFU/mL, respectively; *p* = 0.00094). Additionally, while the I group liquid media titer continued to increase significantly between days 2 and 3 (*p* = 0.0019), the I+T group liquid media titer only slightly increased between these days, with insignificant changes in liquid media titer overall across days 0–3 (*p* = 0.1309). Peak mean BoHV-1 titers for both corneal button and liquid media samples of the I group (corneal button—day 3, 1.14 ± 0.17 × 10^6^ PFU/mL; liquid media—day 3, 3.84 ± 0.11 × 10^6^ PFU/mL) were significantly higher than the initial concentration of virus used to infect each CSR (3 × 10^5^ PFU/5 mL = 6 × 10^4^ PFU/mL), confirming that the CSR tissue was able to support active viral replication during the study period. Figure 4C depicts representative histological changes (epithelial attenuation and disorganization, squamous exfoliation, cellular condensation, heterogenous nuclear chromatin, and eosinophilic cytoplasmic inclusions) occurring late in the culture period, consistent with mild tissue degeneration in both the I and I+T groups.

#### 3.3.2. Corneal Histopathology

When assessing objective corneal measurements amongst and within groups during the study period, no significant differences in epithelial or stromal thickness were observed (*p* = 0.1649 and 0.5119, respectively) amongst groups, though there was a significant increase in epithelial cell density across all treatment groups on day 3 (6.04 ± 0.18 cells/100 μm^2^) relative to days 1 (4.92 ± 0.21 cells/100 μm^2^, *p* = 0.0038) and 2 (5.30 ± 0.18 cells/100 μm^2^, *p* = 0.0469). No significant differences in histologic grading of epithelial stratification or stromal organization were noted between or amongst individual treatment groups throughout the study.

## 4. Discussion

The results of the present study suggest that cidofovir is an efficacious inhibitor of BoHV-1 replication in ocular tissues and warrants further investigation as a treatment for BoHV-1-associated ocular disease in cattle. This drug belongs to a family of nucleoside analogue agents that inhibit viral replication by truncating nucleotide strands during viral DNA production. They are the primary means of treating herpes simplex keratitis in human patients [37] and have been studied extensively in vitro and in vivo for use against other herpesviruses causing ophthalmic disease in domestic animal species, particularly cats [10,11,12,14,15,18,19,38,39,40,41]. Cidofovir inhibition of BoHV-1 has been studied previously, with Gilliam and Field calculating an in-vitro IC_50Number_ of 10 μg/mL (35.8 μM) [23], which is very similar to our independently calculated value described here (39.7 μM). These authors also observed milder ocular discharge and periocular swelling with both prophylactic and therapeutic single subcutaneous cidofovir injections (20 mg/kg) following intranasal BoHV-1 challenge [23]. A study of cidofovir in a cationic lipid vehicle also confirmed viral replication inhibition of BoHV-1 in MDBK cells [26].

In-vivo efficacy of cidofovir has been documented for treatment of both feline herpesvirus-1 and canine herpesvirus-1 ocular infections [12,18,39]. This drug is unique amongst nucleoside analogues in that it exhibits a prolonged duration of action (and therefore requires less frequent administration) attributed to persistence of its intracellular metabolites—specifically cidofovir-phosphate-choline—which may serve as a reservoir of metabolically active viral inhibition [42]. This property would be particularly advantageous for cattle, which are susceptible to herpesvirus recrudescence and more severe clinical signs due to stressors like handling and transportation [43]. For this reason, cidofovir represents a promising agent in this species whose extended effects may reduce or obviate the need for frequent application of topical ophthalmic medication. Of course, the logistical difficulties of topical ophthalmic treatment in cattle merit further investigation into alternate modes of drug delivery. For example, a subconjunctival penciclovir implant was recently developed for the treatment of herpetic keratoconjunctivitis in cats [40], which may also prove to be a useful strategy for extended drug release in cattle that can only be handled infrequently.

In-vivo clinical trials using ophthalmic formulations of cidofovir would be needed for assessment of tolerability, systemic absorption, and associated pharmacokinetic data in cattle. These studies must be performed to establish meat and milk withholding times before clinical use can be recommended in food animal species intended for human consumption [37]. Administration of a single 20 mg/kg subcutaneous dose of cidofovir in a calf subsequently challenged with intranasal BoHV-1 did not result in any serum or urine biochemistry abnormalities suggestive for acute organ damage or malfunction, but no pharmacokinetic or pharmacodynamic data were reported [23]. Since use of a 0.5% topical solution has been well tolerated in cats with no observed systemic effects [39], systemic absorption of ophthalmic cidofovir is likely to be minimal in cattle. Moreover, the dilutional effects of the greater tear film volume [44] and the larger dimensions of the bovine ocular surface [45] may potentially decrease the likelihood for local effects or systemic absorption of a similar concentration of medication delivered to the bovine eye. Local ocular side effects attributed to topical administration of cidofovir have been limited to blepharoconjunctivitis in humans [46] and dogs [18], punctate keratitis in rabbits [47], and lacrimal punctal stenosis in humans and rabbits [48,49]. Cytotoxicity for a given compound is expected to be cell- and host-dependent; for example, in-vitro cytotoxicity of cidofovir to feline corneal epithelial cells has been described to be low [12]. Given the relatively limited in-vitro cytotoxicity to bovine cells observed here, it is expected that ophthalmic administration in cattle would be similarly well tolerated with few side effects in vivo. Future studies of BoHV-1 antiviral inhibition should seek to establish a toxicity index (e.g., half maximal toxic concentration divided by half maximal inhibitory concentration, similar to that described by Babiuk et al. [21]) by including a broader range of drug concentrations in cytotoxicity assays to guide selection of an appropriate dose for in-vivo use. In this instance, post-hoc calculation from cytotoxicity data using a curve fit method like that described for IC_50_ calculation yielded the following TD_50_ and toxicity index (TI) values: cidofovir—D_50_ = 103 μM (TI = 57.2); ganciclovir—TD_50_ = 5972 μM (TI = 21.6); idoxuridine—TD_50_ = 23.5 μM (TI = 14.0); and trifluridine—TD_50_ = 1.04 μM (TI = 9.54). The higher toxicity index for cidofovir reflects lower relative cytotoxicity and further supports the notion that cidofovir was the most potent non-cytotoxic agent tested, although more rigorous cytotoxicity assay design would be needed to confirm these calculations.

Previous studies have described the effects of other nucleoside analogue medications on BoHV-1. We chose to assess cytotoxicity of the antiviral compounds using MDBK cells. Although the origin of MDBK cells is non-ocular, this is a well-characterized, readily available, and reliable cell line. Similar assessments of ocular antivirals for treatment of veterinary herpesviruses have also used non-ocular cell lines, presumably for the same reasons [10,11,13,16,18,19]. An in-vitro comparison of a large group of nucleoside analogue agents determined (E)-5-(2-bromovinyl)-2′-deoxyuridine (BVdU) and trifluridine exhibited superior BoHV-1 antiviral potency versus cytarabine, idoxuridine, foscarnet, phosphonoacetic acid, vidarabine, and acyclovir [21]. Though no objective measurement of cytotoxicity was included in this study, trifluridine exhibited a lower therapeutic index based upon visual observation of nonviable Georgia Bovine Kidney cells in vitro, suggesting greater toxicity than BVdU, and was therefore not evaluated in vivo [21]. These results reflect our findings of visible MDBK cell cytotoxicity (cell rounding, poor adhesion) at low concentrations in vitro. Together, these results suggest that trifluridine should be avoided for use in cattle despite its high BoHV-1 inhibitory potency due to a higher likelihood for adverse effects of ocular irritation, which is often observed in cats after topical application [50]. Ultimately, the previous study found therapeutic oral administration of BVdU had no effect on the level of viral shedding, clinical signs, or susceptibility to secondary bacterial infection in calves challenged with intranasal BoHV-1 infection despite its in-vitro potency [21]. Relatively poor activity of acyclovir and ganciclovir against BoHV-1 compared to other antiviral compounds (e.g., foscarnet) have been described in prior reports [22,24].

To the authors’ knowledge, this study is the first to describe an ex-vivo model of BoHV-1 keratitis. Descriptions of herpesvirus keratitis models in other species emphasize the maintenance of a viable corneal epithelium, which is the primary site of virus replication in the cornea [27], and previous reports have characterized epithelial degeneration following canine and feline herpesvirus infections that is mitigated by nucleoside analogue antiviral treatment [29,35]. In contrast, we did not observe significant differences in epithelial thickness amongst either uninfected control corneas or BoHV-1-infected corneas with or without cidofovir treatment throughout the course of our study. The BoHV viral load selected to inoculate CSRs was derived from a previous report of ex-vivo feline herpesvirus modeling conducted over 48 h of culture that described significant differences in epithelial parameters between untreated and treated corneas following infection during this time frame [29]. Extending the duration of the culture beyond three days might have resulted in more notable differences with an expected smaller reduction in epithelial thickness in cidofovir-treated corneas (I+T group) relative to untreated, BoHV-1 infected corneas (I group). Though there was a significant increase in epithelial cell density on day 3 (6.0 ± 0.18 cells/100 μm^2^) relative to days 1 (4.92 ± 0.21 cells/100 μm^2^) and 2 (5.30 ± 0.18 cells/100 μm^2^), the explanation for this finding is unclear but may be attributed to random effect within the relatively small sample size or potential tangential cuts through the epithelium during histologic processing. Harman et al. [35] noted a significant decrease in epithelial cell density (measured similarly as nuclei counted within the epithelium of histologic specimens) in the first 10 days of ex-vivo canine corneal culture that was attributed to potential epithelial cell expansion from mechanical stress release. Extending our culture period beyond three days may have resulted in a similar trend. Overall, the fact that viral replication was sustained over a three-day period to permit recovery of viable virus particles with minimal tissue degradation supports the use of this ex-vivo corneal model as a mode of replacement and reduction in animal research [27].

The present study serves as the basis for future investigations of BoHV-1 ocular infection and antiviral inhibition. With continued optimization, the model might prove useful for assessment of other herpesviruses implicated in bovine ocular disease (BoHV-4) [51,52] or coinfection with both BoHV-1 and other etiologic agents (e.g., *M. bovis*) to better understand the contribution of co-infections in the pathophysiology of IBK [7,8,9,53]. Ex-vivo studies can supplement clinical investigation to observe the interaction of these pathogens at the tissue level so that more effective treatments and management strategies can be ultimately devised to combat debilitating and costly infectious ocular disease in cattle.

## 5. Conclusions

The results of this study suggest that cidofovir is an effective inhibitor of BoHV-1 in both in-vitro and ex-vivo settings, with only minimal associated cytotoxicity to bovine tissues. Trifluridine is also a potent inhibitor of BoHV-1 replication in vitro but exhibits significantly greater cytotoxicity to bovine cells, while idoxuridine and ganciclovir have relatively poor BoHV-1 replication inhibition in vitro. We propose that cidofovir merits further investigation as an agent to address herpetic keratoconjunctivitis in cattle. The ex-vivo bovine corneal model described here is a valuable tool for modeling BoHV-1 infection of ocular tissues and may be employed in future assays to refine antiviral dosing and delivery recommendations.

## Figures and Tables

**Table 1:** Half-maximal inhibitory concentrations for nucleoside analogue antiviral agents against BoHV-1, determined using curve fit calculation.

Drug	IC_50Number_ (µM)	IC_50Area_ (µM)	Number of Concentrations Tested for Curve (*n*)
Trifluridine	0.517	0.110	6
Cidofovir	39.7	1.80	31
Idoxuridine	1.32 × 10^4^	23.5	36
Ganciclovir	2.12 × 10^4^	277	21

## Data Availability

Data is either contained within the article or available upon request.
